# A Novel Tool to Mitigate By-Catch Mortality of Baltic Seals in Coastal Fyke Net Fishery

**DOI:** 10.1371/journal.pone.0127510

**Published:** 2015-05-18

**Authors:** Sari M. Oksanen, Markus P. Ahola, Jyrki Oikarinen, Mervi Kunnasranta

**Affiliations:** 1 Department of Biology, University of Eastern Finland, Joensuu, Finland; 2 Natural Resources Institute Finland, Turku, Finland; 3 Perämeren Kalatalousyhteisöjen Liitto ry, Oulu, Finland; Bangor University, UNITED KINGDOM

## Abstract

Developing methods to reduce the incidental catch of non-target species is important, as by-catch mortality poses threats especially to large aquatic predators. We examined the effectiveness of a novel device, a “seal sock”, in mitigating the by-catch mortality of seals in coastal fyke net fisheries in the Baltic Sea. The seal sock developed and tested in this study was a cylindrical net attached to the fyke net, allowing the seals access to the surface to breathe while trapped inside fishing gear. The number of dead and live seals caught in fyke nets without a seal sock (years 2008–2010) and with a sock (years 2011–2013) was recorded. The seals caught in fyke nets were mainly juveniles. Of ringed seals (*Phoca hispida botnica*) both sexes were equally represented, while of grey seals (*Halichoerus grypus*) the ratio was biased (71%) towards males. All the by-caught seals were dead in the fyke nets without a seal sock, whereas 70% of ringed seals and 11% of grey seals survived when the seal sock was used. The seal sock proved to be effective in reducing the by-catch mortality of ringed seals, but did not perform as well with grey seals.

## Introduction

Fisheries worldwide capture several non-target species as incidental by-catch. Populations of many large aquatic predators, such as marine mammals and sharks are especially vulnerable to by-catch mortality in consequence of their slow reproductive rates [1]. Mitigation of by-catch mortality has therefore become increasingly important in both fisheries management [2] and wildlife conservation [3]. However, the interactions between aquatic predators and fisheries can be complex, and in particular, many seal species cause losses to fish catches and damage to fishing gear [4,5]. Developing fishing methods can provide means to mitigate both aspects of the seal-fisheries conflict. For example, physical barriers mounted on the fish traps, such as wire grids, prevent seals from entering the trap [6–8]. Alternatively, exclusion devices that facilitate the seals’ exit from the fishing gear [9] and techniques that trap seals alive inside the gear have also been developed [10].

**Fig 1:**
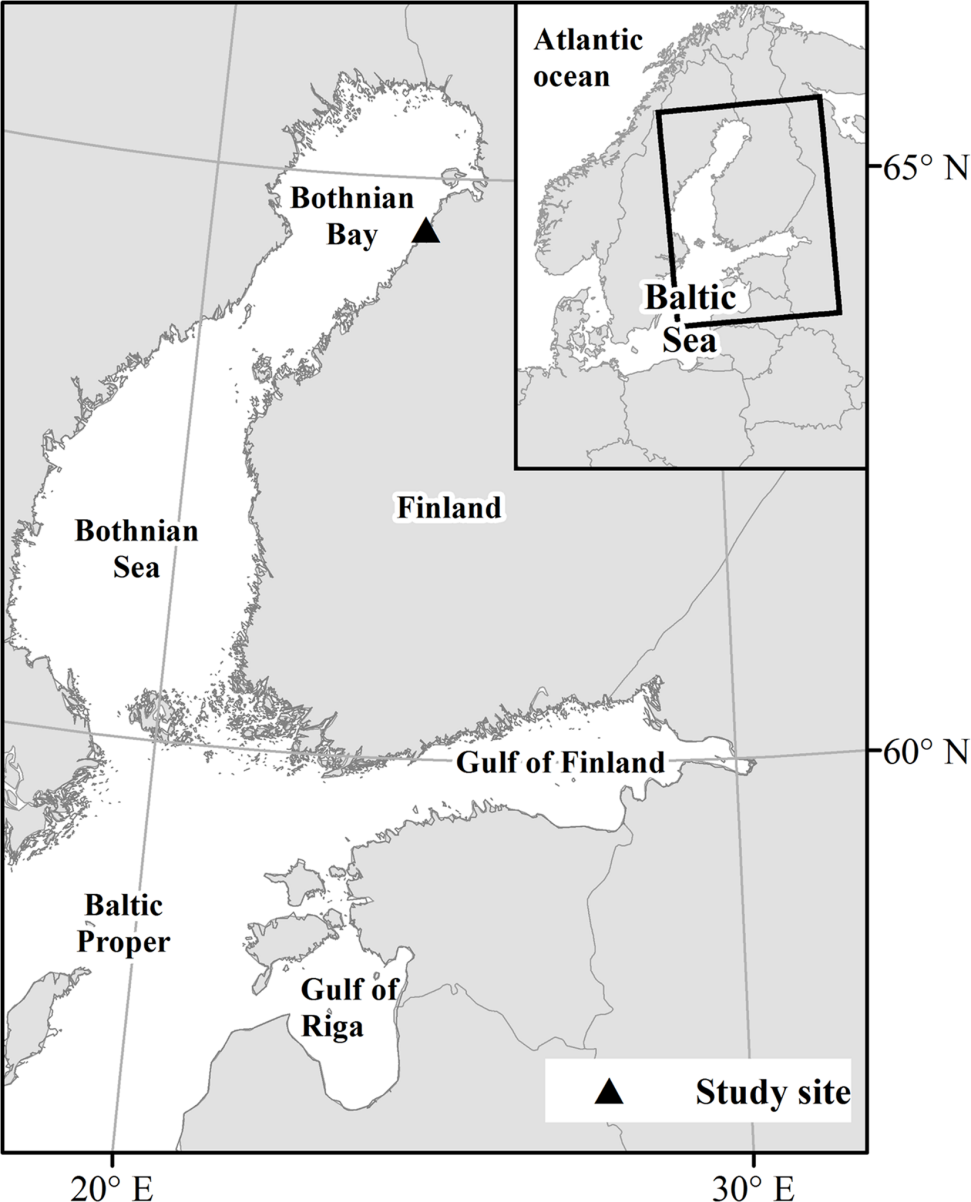
The Baltic Sea.

The Baltic grey seal (*Halichoerus grypus*) and ringed seal (*Phoca hispida botnica*) have had significant interactions with human activities. Both populations declined drastically to only about 5000 seals in the 1970s as a result of excessive hunting and reproductive disorders caused by environmental pollution. The seal populations have been recovering, but the present number of grey seals (census size: 30 000 seals) is still less than half and ringed seals (13 000 seals) less than one tenth of the estimated historical abundance at the beginning of the 20^th^ century [11–13]. The recovery of these populations has led to an increase in seal-induced losses to coastal fisheries, which has had negative effects on attitudes towards the conservation of seal populations [14]. The Baltic grey seal in general causes more losses to fisheries than the ringed seal [6,15]. However, increasing numbers of ringed seals are assumed to cause substantial catch losses in the Bothnian Bay, the northernmost part of the Baltic Sea (Fig 1) [16]. The hunting of seals has become a key action for the mitigation of damage in the northern Baltic Sea [14,17]. In addition to hunting, by-catch mortality may be another significant component of the anthropogenic mortality of seal populations. Estimated annual by-catch of the Baltic grey seal is in the order of 2000 animals or more [18] but for ringed seals the magnitude is unknown. An information gap regarding by-catch and its mitigation methods in coastal and small scale fisheries has been recognized globally [19]. Also in the Baltic Sea broader knowledge of by-catch is of great importance for sustainable fishery and population management [20].

Considerable efforts have been put into research on interaction between Baltic grey seal and fisheries [10,14,17,18,21,22] and on the development of seal-proof fishing gear. For example, trap nets with modified fish chambers, i.e. pontoon traps, are reported to reduce grey seal-induced losses to fisheries and simultaneously to reduce by-catch [8,23]. However, relatively little attention has been paid to estimating and reducing the by-catch mortality of seals in other stationary gears. In addition, there is little information on the interaction between ringed seals and fisheries, not only in the Baltic Sea [24], but also globally. Methods for reducing interactions of Baltic ringed seals and coastal fishery are of great importance, as climate change can be expected to further threaten the population growth of this ice-breeding species, especially in the southern breeding areas, where the stocks have recovered slowly, if at all [12]. In this study we tested the effectiveness of a new type of pinniped by-catch reduction device for fyke nets, called a seal sock. We also examined the demographic properties, such as age and sex distribution, of the by-caught seals.

## Methods

Around 70% of the Baltic ringed seal population is found in the Bothnian Bay (Fig 1), and grey seal is also encountered in this region. Coastal fyke net fishery in the area targets whitefish (*Coregonus lavaretus*), vendace (*C*. *albula*), Atlantic salmon (*Salmo salar*) and brown trout (*S*. *trutta*) in particular. Various modifications of fyke nets are used, but in general they are large, bottom-anchored fishing gear that consists of a leader net and wings guiding the fish through the middle chamber into the fish chamber. Seals entering the chambers may not find their way out and drown, as the chambers are usually submerged (approximately 2 m from the surface). The seal sock is a by-catch reduction device that is designed to enable seals to have access to the surface to breathe while trapped inside the fyke net. The sock was initially developed and constructed by a Finnish fish trap manufacturer (Ab Scandi Net Oy).

**Fig 2:**
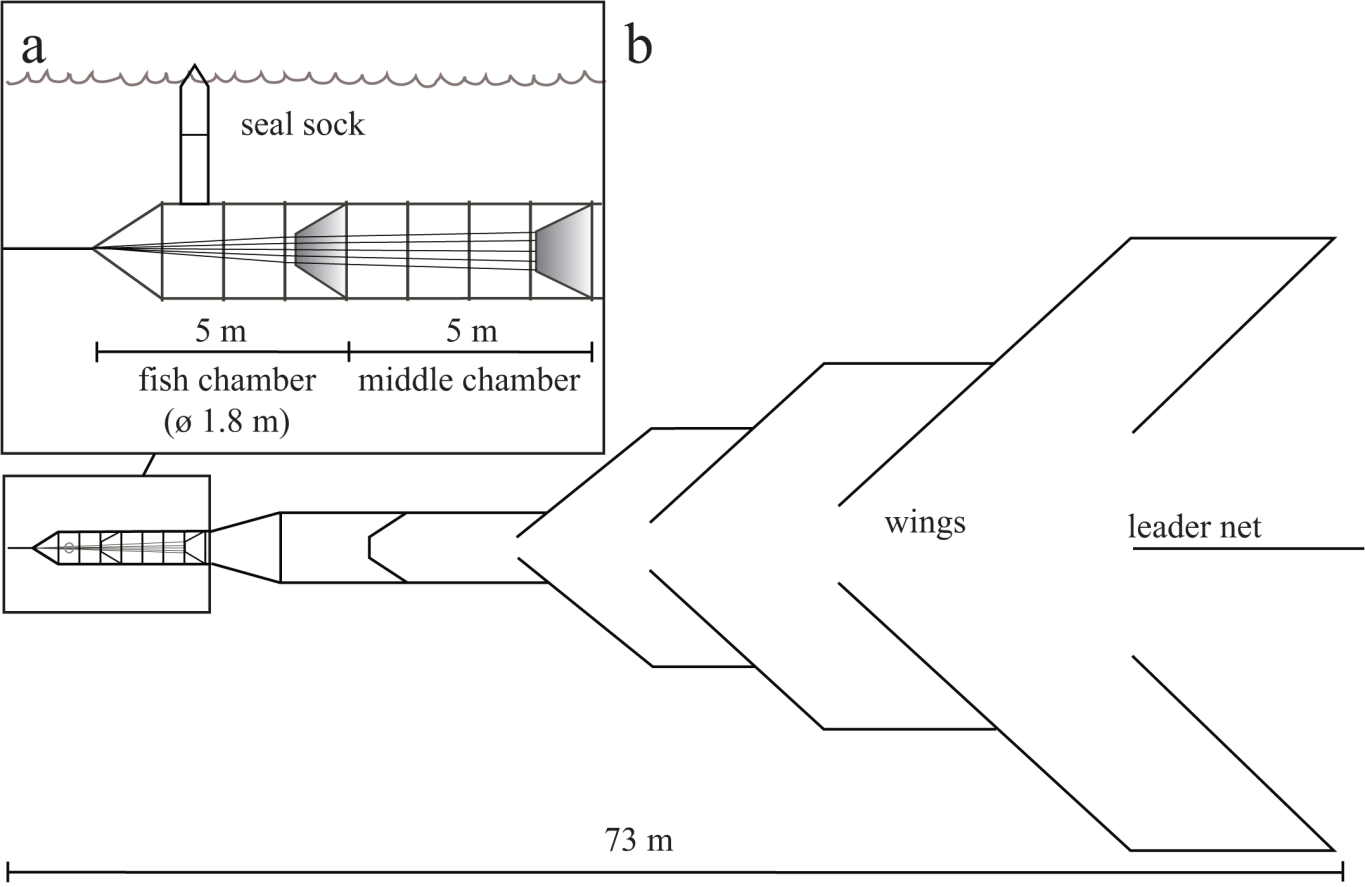
A type of by-catch reduction device, a seal sock, is attached to the fish chamber of a fyke net allowing the seal to have access to the surface. (A) A side view of a seal sock and chambers. (B) A fyke net (seen from above) consists of a leader net, wings and chambers.

We developed a modification of the seal sock, comprising a cylindrical net (diameter 0.7 m, length 2.5–4.0 m) made of strong seal-proof netting (Dyneema, mesh size 30 mm). It was attached to the roof of the fish chamber in a fyke net (Fig 2A). The sock was fitted with one or two hoops, to keep its shape, and a small float. To examine the effectiveness of the seal sock in reducing by-catch, we compared the number of seals caught alive in a set of fyke nets between years when the socks were not used (2008–2010) and when they were used (2011–2013). Annually, between May and October–November, a commercial fisherman fished with 4–6 fyke nets within the same fishing area in the Bothnian Bay (64°32N, 24°19E, Fig 1). The fyke nets comprised a leader net (mesh size 200–300 mm), wings (60–100 mm) and two cylindrical chambers (30–35 mm, Dyneema, Fig 2B). Fishing effort was determined based on the fisherman’s logbooks and was relatively same between both periods (2106 days for years 2008–2010 and 1767 days for years 2011–2013).

The fisherman kept a logbook on the number, species and capture date of by-caught seals. During the testing of the sock in the years 2011–2013, the fisherman also recorded the sex and weight (kg) or age class (juveniles and adults) of the by-caught seals. Researchers took part to the field work (tagging live animals and taking samples from dead ones) during years 2011–2013 and they often had possibility to verify the reliability of the data. Although the fishermen’s motivation to report by-catch has generally been low in Finland [18], the volume of by-catch reported in this study is high and comparable between the two periods (2008–2010 and 2011–2013). It is therefore likely that the volumes of by-catch detected in our study are not underrated. Weighed seals were further divided into age classes on the basis of an age-weight database (Natural Resources Institute Finland). Weighed ringed seals with a body weight > 50 kg were classified as adults (estimated age ≥ 4 years). Grey seal males with a body weight > 92 kg and females > 65 kg were categorized as adults (estimated age ≥ 5 years). We also used the seal socks for live-capturing ringed seals for satellite tagging (Oksanen et al., unpublished). We tested the effect of the seal sock on survival with Fisher’s exact test. The effect of weight and capture month on survival of ringed seals when the sock was in use (2011–2013) was tested with binary logistic regression. Effects of weight and month on the grey seal survival were not tested due to the small sample size. We conducted statistical testing with IBM SPSS Statistics 20 software.

### Ethics Statement

The use of a seal sock and animal handling was permitted by the game authorities (permit no. 2011/00082 and 2013/00197) and the Animal Experiment Board of Finland (no. ES AVI/1114/04.10.03/2011).

## Results

A total of 135 seals (30 live and 105 dead) were caught (4–6 fyke nets annually) during the study years in 2008–2013 (S1 Dataset). Of all the by-caught seals, 103 (76%) were ringed and 32 (24%) grey seals. During the years 2011–2013, 95% of the ringed (n = 40) and 67% of the grey seals (n = 18) were juveniles (Table 1). The mean weight of the by-caught and weighed ringed seals (n = 33) was 36 kg (SD 11). The weight of the ringed seals visiting fyke nets increased towards autumn (linear regression, R^2^ = 0.469, F = 27.3, p<0.001; Table 1): ringed seals by-caught and weighed in May (n = 4) had a mean weight of 19 kg (SD 9) (corresponding to young-of-the-year), whereas individuals by-caught in October–November (n = 7) weighed on average 44 kg (SD 10). The sex-ratio of the ringed seals was even (49% males and 51% females), but for the grey seals it was biased towards males (71% males and 29% females; Table 1). In fact, while the sex distribution of juvenile grey seals (n = 12) was even and they were caught mostly during summer (8 caught in May-July), the adults (n = 6) were mostly males (5 males, 1 unknown) and mostly captured during the autumn (5 caught in August-November: Table 1).

**Table 1:** Monthly variation in numbers, age and sex distribution and survival of the by-caught seals in fyke nets equipped with the seal sock.

Month	Ringed seals					Grey seals				Total
	Total	Juveniles/ adults	Males/ females	Alive/ dead	Mean weight	Total	Juveniles/ adults	Males/ females	Alive/ dead	
May	6	6/0	3/3	2/4	19 ± 9	3	3/0	1/2	0/3	**9**
June	1	1/0	1/0	0/1	na	3	2/1	3/0	0/3	**4**
July	3	3/0	2/1	2/1	24 ± 8	3	3/0	1/2	0/3	**6**
Aug	4	4/0	2/2	4/0	34 ±8	2	1/1	2/0	0/2	**6**
Sept	16	15/1	7/9	13/3	38 ± 8	4	2/2	4/0	1/3	**20**
Oct	8	7/1	3/4^a^	5/3	45 ± 12	1	1/0	0/1	1/0	**9**
Nov	2	2/0	1/1	2/0	43 ± 1	2	0/2	1/0^a^	0/2	**4**
**Total**	**40**	**38/2**	**19/20**	**28/12**	**36 ± 11**	**18**	**12/6**	**12/5**	**2/16**	**58**

In years 2011–2013, annually 4–6 fyke nets equipped with the seal sock were set out for fishing in the Bothnian Bay. Monthly mean weight (kg) ± SD for ringed seals is reported for a total of 33 weighed individuals.

^a^ Gender for one seal not recorded

**Fig 3:**
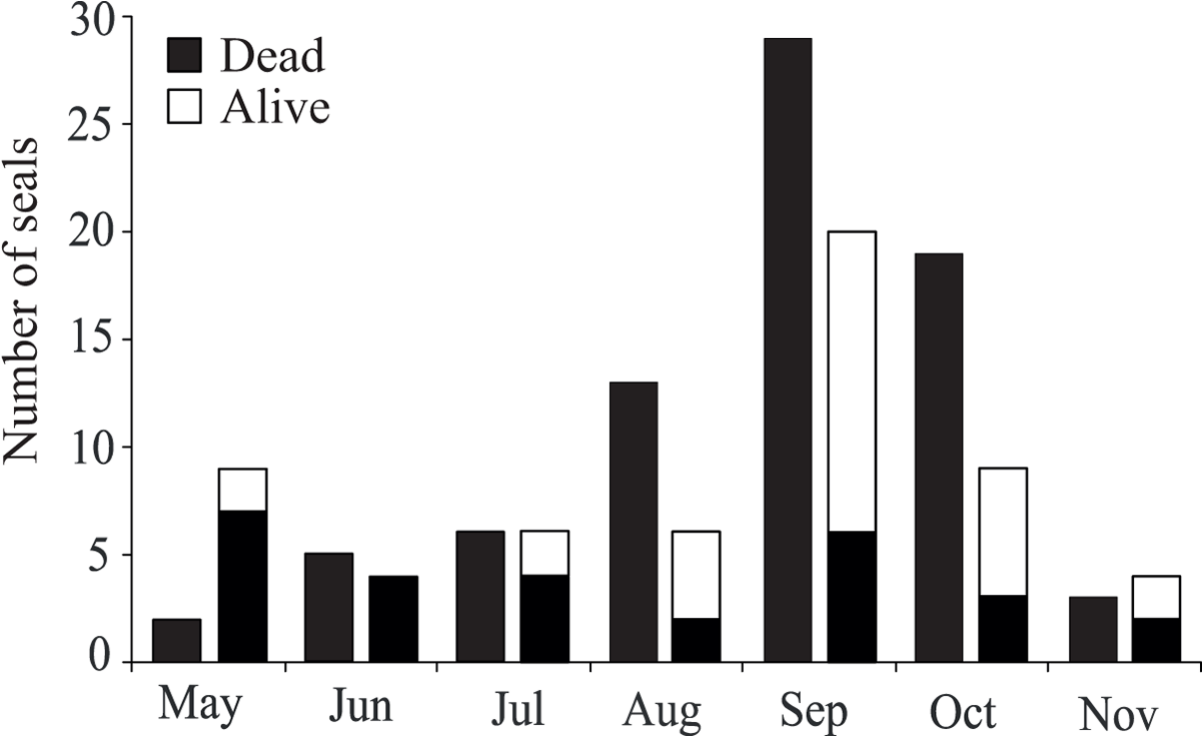
Monthly variation in the number of by-caught seals in fyke nets (4–6 fyke nets annually). First bars: fyke nets without a seal sock (years 2008–2010). Second bars: fyke nets with a seal sock (2011–2013).

Overall, the survival of seals in the fyke nets increased when the sock was used (Fisher’s exact test, p<0.001). Altogether 77 dead seals were by-caught during the years when the seal sock was not used (2008–2010), whereas 30 live and 28 dead seals were caught when the seal sock was used (2011–2013) (Fig 3). The seal sock increased survival of ringed seals (p<0.001): of all caught ringed seals (n = 40), 83% found their way to the sock and 70% remained alive. However, the survival of grey seals was not increased (p = 0.308): only 17% of the grey (n = 18) seals found way to the sock, and 11% remained alive. The ringed seals that did not find their way into the sock were mostly small pups (5/7) having a mean weight of 16 kg (SD 9) and they were mostly captured in early summer. In fact, capture month was the only statistically significant predictor of ringed seal survival in logistic regression (model summary: χ^2^(1) = 4.210, p = 0.040, Nagelkerke R^2^ = 0.142; parameter *month*: p = 0.048, coefficient = –0,405). By contrast, all the by-caught adult grey seals (n = 6) were caught in the middle chamber, while 8 out of 12 of juveniles were either in the fish chamber or in the sock. However, only 3 out of 8 grey seals reaching the fish chamber found their way into the sock.

## Discussion

The seal sock proved to be effective in reducing the by-catch mortality of ringed seals in fyke nets, but it did not perform as well with grey seals. According to our pilot test, the seal sock also seemed to work well in a so-called bottom fyke net (Flex R, Ab Scandi Net Oy), which we tested during the summer of 2013, and all of the captured seals (7 ringed seals and 1 grey seal) survived in the sock. Although the use of the seal sock increased survival of ringed seals in general, small pups caught during early summer survived poorly despite the sock. On the contrary, adult grey seals were by-caught only in the middle chamber, and it is likely that they could not reach the fish chamber through the narrow passage between the chambers (diameter 0.3 m). To overcome this problem, a sock could be inserted also into the middle chamber. However, only 3 out of 8 grey seals that reached the fish chamber found their way into the sock, which may indicate behavioral differences between the species. Ringed seals keep open breathing holes in the sea ice, which may be several meters thick in the Arctic [25], and swimming upwards in a narrow funnel might, therefore, be more typical behavior for them. In addition to reducing by-catch, the seal sock has potential applications in capturing seals alive and removing individuals repeatedly visiting the fishing gear. When the seal sock is used only for by-catch mitigation, it could have an opening at the surface enabling seals to climb out of the gear, as exclusion devices are usually designed to enable rapid exit of the animal from the fishing gear.

Our results illustrate that the majority of seals by-caught in fyke nets are young seals of both genders. This was especially evident in ringed seals, of which 95% were juveniles and equally of both genders, but also among grey seals most of the by-caught individuals were juveniles (67%). The dominance of juveniles in by-catch could to some extent be explained by their naivety, as juveniles of many seal species are more vulnerable to being by-caught [26,27]. Ringed seal pups are especially prone to get caught in fishing gear just after weaning in the early summer [28]. We also observed lower survival of ringed seals during early summer compared to autumn, and our results indicated that survival in the seal sock was more dependent on naivety of the young-of-the-year than weight of the seals. Although the overall sex ratio of grey seals was biased towards males, young grey seals of both sexes were by-caught especially in spring, and adult males in autumn. A similar temporal trend in by-caught grey seals has been reported in previous studies [29,30]. A strong male dominance has also been observed in grey seals visiting pontoon traps [10,21,22,31].

Our study indicates that by-catch particularly increases juvenile mortality, which may not reduce population growth and viability as much as a similar increase in adult—especially female—mortality would [32]. Nevertheless, by-catch mortality may constitute another significant source of anthropogenic mortality in the grey seal population, whose annual hunting pressure in the Finnish sea area was around 5% of the total population [33], and should therefore be taken into account in the population management. By-catch mortality also increases total mortality of the ringed seal population and mitigating it may become increasingly important for population management, as the population growth of this ice-dependent species is predicted to decrease due to climate change [12,34].

Knowledge of the by-catch mortality and its mitigation methods are important aspects of the sustainable management of seal populations. Reducing incidental mortality is also becoming increasingly important measure for the fisheries management to respond to growing demands for sustainable seafood. Several ecolabels take by-catch reduction into account [2]. For example ecolabel of the Marine Stewardship Council (MSC) is granted to fisheries that follow the sustainable fishery standards of the MSC. Our study introduces a novel and inexpensive tool, a seal sock, for mitigating by-catch of seals in coastal fyke net fisheries. In addition, the seal sock provides a practical and ethical method for the selective removal of seals repeatedly visiting fyke nets in areas where high seal abundance causes substantial losses to fisheries. It can also be used in scientific studies where seals need to be captured alive, for example telemetry studies.

## Supporting Information

S1 Dataset Details of seals by-caught in fyke nets (4–6 fyke nets annually) during the years 2008–2013. The fyke nets were set out for fishing without a seal sock in the years 2008**–**2010 and with a seal sock in 2011**–**2013 in the Bothnian Bay. Species: Phb = Baltic ringed seal, *Phoca hispida botnica*; Hg = grey seal, *Halichoerus grypus*. Location of the seal in a fyke net: sock/ middle chamber/ fish chamber/ wings. na = not available
(PDF)
