# Supplementary material for: A Novel Tool to Mitigate By-Catch Mortality of Baltic Seals in Coastal Fyke Net Fishery
Source: PLoS One. 2015 May 18;10(5):e0127510. doi: 10.1371/journal.pone.0127510 (PMC4436298; doi:10.1371/journal.pone.0127510)
Supplement: S1 Dataset — The fyke nets were set out for fishing without a seal sock in the years 2008–2010 and with a seal sock in 2011–2013 in the Bothnian Bay. Species: Phb = Baltic ringed seal, Phoca hispida botnica; Hg = grey seal, Halichoerus grypus. Location of the seal in a fyke net: sock/ middle chamber/ fish chamber/ wings. na = not available (PDF) [file pone.0127510.s001.pdf]

Year	Month	Date	Species (Phb/Hg)	Gender (f/m)	Weight (kg) or age class (juvenile/adult)	Status (dead/alive)	Location in the fyke net
2008	5	20.5.2008	Phb	na	na	dead	na
2008	5	30.5.2008	Hg	na	na	dead	na
2008	6	16.6.2008	Phb	na	na	dead	na
2008	6	20.6.2008	Phb	na	na	dead	na
2008	6	20.6.2008	Phb	na	na	dead	na
2008	6	27.6.2008	Phb	na	na	dead	na
2008	6	28.6.2008	Phb	na	na	dead	na
2008	7	25.7.2008	Hg	na	na	dead	na
2008	7	29.7.2008	Phb	na	na	dead	na
2008	7	29.7.2008	Hg	na	na	dead	na
2008	8	11.8.2008	Phb	na	na	dead	na
2008	8	11.8.2008	Phb	na	na	dead	na
2008	8	16.8.2008	Hg	na	na	dead	na
2008	8	21.8.2008	Phb	na	na	dead	na
2008	8	27.8.2008	Phb	na	na	dead	na
2008	8	27.8.2008	Hg	na	na	dead	na
2008	8	29.8.2008	Phb	na	na	dead	na
2008	8	29.8.2008	Phb	na	na	dead	na
2008	9	5.9.2008	Phb	na	na	dead	na
2008	9	5.9.2008	Phb	na	na	dead	na
2008	9	11.9.2008	Phb	na	na	dead	na
2008	9	11.9.2008	Phb	na	na	dead	na
2008	9	11.9.2008	Phb	na	na	dead	na
2008	9	11.9.2008	Phb	na	na	dead	na
2008	9	11.9.2008	Phb	na	na	dead	na
2008	9	14.9.2008	Phb	na	na	dead	na
2008	9	19.9.2008	Phb	na	na	dead	na
2008	10	9.10.2008	Hg	na	na	dead	na
2008	10	9.10.2008	Hg	na	na	dead	na
2008	10	11.10.2008	Phb	na	na	dead	na
2008	10	11.10.2008	Phb	na	na	dead	na
2008	10	15.10.2008	Phb	na	na	dead	na
2009	7	3.7.2009	Hg	na	na	dead	na
2009	7	19.7.2009	Phb	na	na	dead	na
2009	8	25.8.2009	Phb	na	na	dead	na
2009	8	25.8.2009	Phb	na	na	dead	na
2009	9	3.9.2009	Phb	na	na	dead	na
2009	9	3.9.2009	Hg	na	na	dead	na
2009	9	6.9.2009	Phb	na	na	dead	na
2009	9	6.9.2009	Hg	na	na	dead	na
2009	9	13.9.2009	Hg	na	na	dead	na
2009	9	18.9.2009	Phb	na	na	dead	na
2009	9	25.9.2009	Phb	na	na	dead	na
2009	9	25.9.2009	Hg	na	na	dead	na
2009	10	7.10.2009	Phb	na	na	dead	na
2009	10	7.10.2009	Phb	na	na	dead	na

2009	10	11.10.2009	Phb	na	na	dead	na
2009	10	18.10.2009	Phb	na	na	dead	na
2009	10	20.10.2009	Phb	na	na	dead	na
2009	10	24.10.2009	Phb	na	na	dead	na
2009	10	27.10.2009	Phb	na	na	dead	na
2009	10	29.10.2009	Phb	na	na	dead	na
2009	11	4.11.2009	Phb	na	na	dead	na
2009	11	4.11.2009	Phb	na	na	dead	na
2009	11	5.11.2009	Phb	na	na	dead	na
2010	7	2.7.2010	Phb	na	na	dead	na
2010	8	13.8.2010	Phb	na	na	dead	na
2010	8	13.8.2010	Phb	na	na	dead	na
2010	8	16.8.2010	Phb	na	na	dead	na
2010	9	10.9.2010	Hg	na	na	dead	na
2010	9	12.9.2010	Phb	na	na	dead	na
2010	9	12.9.2010	Phb	na	na	dead	na
2010	9	14.9.2010	Phb	na	na	dead	na
2010	9	14.9.2010	Phb	na	na	dead	na
2010	9	14.9.2010	Phb	na	na	dead	na
2010	9	17.9.2010	Phb	na	na	dead	na
2010	9	17.9.2010	Phb	na	na	dead	na
2010	9	17.9.2010	Phb	na	na	dead	na
2010	9	21.9.2010	Phb	na	na	dead	na
2010	9	25.9.2010	Phb	na	na	dead	na
2010	9	28.9.2010	Phb	na	na	dead	na
2010	10	5.10.2010	Hg	na	na	dead	na
2010	10	7.10.2010	Phb	na	na	dead	na
2010	10	18.10.2010	Phb	na	na	dead	na
2010	10	19.10.2010	Phb	na	na	dead	na
2010	10	21.10.2010	Phb	na	na	dead	na
2010	10	21.10.2010	Phb	na	na	dead	na
2011	5	26.5.2011	Hg	f	juvenile	dead	middle chamber
2011	6	4.6.2011	Hg	m	juvenile	dead	wings
2011	8	20.8.2011	Phb	m	40	alive	sock
2011	9	2.9.2011	Phb	f	38	alive	sock
2011	9	8.9.2011	Hg	m	juvenile	dead	fish chamber
2011	9	8.9.2011	Hg	m	juvenile	alive	sock
2011	9	9.9.2011	Phb	f	33	alive	sock
2011	9	22.9.2011	Phb	f	50	alive	sock
2011	9	25.9.2011	Phb	m	35	alive	sock
2011	10	1.10.2011	Phb	m	juvenile	dead	sock
2011	10	1.10.2011	Phb	m	juvenile	dead	fish chamber
2012	5	17.5.2012	Phb	f	17	dead	fish chamber
2012	5	22.5.2012	Phb	f	25	alive	sock
2012	5	23.5.2012	Phb	m	25	alive	sock
2012	5	29.5.2012	Hg	f	juvenile	dead	sock
2012	5	31.5.2012	Hg	m	juvenile	dead	fish chamber
2012	5	25.5.2012	Phb	f	7	dead	fish chamber
2012	6	29.6.2012	Phb	m	juvenile	dead	sock
2012	6	29.6.2012	Hg	m	juvenile	dead	fish chamber

2012	6	29.6.2012	Hg	m	adult	dead	middle chamber
2012	7	6.7.2012	Phb	m	30	alive	sock
2012	7	10.7.2012	Phb	m	18	dead	sock
2012	7	13.7.2012	Hg	f	juvenile	dead	fish chamber
2012	7	14.7.2012	Phb	f	juvenile	alive	sock
2012	7	14.7.2012	Hg	f	juvenile	dead	fish chamber
2012	8	2.8.2012	Phb	f	28	alive	sock
2012	8	12.8.2012	Phb	m	27	alive	sock
2012	8	29.8.2012	Phb	f	42	alive	sock
2012	9	3.9.2012	Phb	m	29	alive	sock
2012	9	3.9.2012	Phb	f	32	dead	sock
2012	9	16.9.2012	Phb	f	54	dead	fish chamber
2012	9	22.9.2012	Phb	f	47	alive	sock
2012	9	24.9.2012	Phb	m	33	alive	sock
2012	9	28.9.2012	Phb	m	24	dead	fish chamber
2012	10	3.10.2012	Phb	f	46	dead	sock
2012	10	6.10.2012	Phb	f	65	alive	sock
2012	10	10.10.2012	Hg	f	juvenile	alive	sock
2012	10	11.10.2012	Phb	f	34	alive	sock
2012	10	15.10.2012	Phb	f	37	alive	sock
2013	5	27.5.2013	Phb	m	juvenile	dead	fish chamber
2013	5	27.5.2013	Phb	m	juvenile	dead	fish chamber
2013	7	11.7.2013	Hg	m	64	dead	middle chamber
2013	7 or 8	na	Hg	m	juvenile	dead	middle chamber
2013	8	16.8.2013	Hg	m	154	dead	middle chamber
2013	9	11.9.2013	Phb	f	37	alive	sock
2013	9	19.9.2013	Phb	m	45	alive	sock
2013	9	19.9.2013	Phb	m	33	alive	sock
2013	9	19.9.2013	Hg	m	112	dead	middle chamber
2013	9	24.9.2013	Phb	m	37	alive	sock
2013	9	24.9.2013	Phb	f	45	alive	sock
2013	9	27.9.2013	Phb	f	30	alive	sock
2013	9	28.9.2013	Hg	m	98	dead	middle chamber
2013	10	1.10.2013	Phb	na	juvenile	alive	sock
2013	10	12.10.2013	Phb	m	43	alive	sock
2013	11	1.11.2013	Hg	m	150	dead	middle chamber
2013	11	2.11.2013	Phb	f	44	alive	sock
2013	11	5.11.2013	Phb	m	42	alive	sock
2013	11	na	Hg	na	adult	dead	middle chamber
